# Atrial flutter catheter ablation in adult congenital heart diseases

**DOI:** 10.1016/j.ipej.2021.06.003

**Published:** 2021-06-19

**Authors:** Victor Waldmann, Francis Bessière, Cristina Raimondo, Alice Maltret, Denis Amet, Eloi Marijon, Nicolas Combes

**Affiliations:** a Adult Congenital Heart Disease Medico-Surgical Unit, AP-HP, Georges Pompidou European Hospital, Paris, France; b Pediatric and Congenital Cardiology Department, AP-HP, Necker Hospital, Paris, France; c Université de Paris, Paris, France; d Louis Pradel Cardiovascular Hospital, Cardiac Electrophysiology Department, Hospices Civils de Lyon, Lyon, France; e Université de Lyon, Lyon, France; f Marie Lannelongue Hospital, Department of Pediatric Cardiology and Congenital Heart Diseases, Centre de Référence Cardiopathies Congénitales Complexes M3C, Le Plessis-Robinson, France; g Department of Pediatric Cardiology and Congenital Heart Diseases, Pasteur Clinic, Toulouse, France

**Keywords:** Atrial flutter, Congenital heart disease, Atrial arrhythmia, Catheter ablation

## Abstract

The important increase in life expectancy of adult patients with congenital heart disease (ACHD) has generated new challenges, including arrhythmias that represent one of the main late complications. Reentrant atrial arrhythmias are by far the main mechanism encountered, and catheter ablation has been now presented as a first-line therapy in this patient population. The number of procedures is expected to continuously increase year after year. The heterogeneity and complexity of phenotypes encountered require these cases to be performed by highly experienced operators, in specialized centers with multidisciplinary competencies. A thorough knowledge and understanding of anatomic specificities, vascular access issues, and main circuits encountered according to underlying phenotype is essential. Acute success rates have significantly improved and are now excellent, but recurrences remain a common issue, with different mechanisms or circuits frequently encountered. Observational data have suggested the interest of systematically targeting all inducible atrial arrhythmias, whether previously documented or not, and a lot of hope and research is based on the prediction of arrhythmia substrate before arrhythmia development by imaging or electroanatomic mapping to deliver a prophylactic patient tailored ablation approach. In this review, we summarize those different points in the most common or distinctive defects to offer a didactic overview of atrial flutter catheter ablation in ACHD patients.

## 1 Introduction

Recent advances in pediatric cardiology, especially surgical techniques, have resulted in an increasing number of patients with congenital heart disease reaching adulthood, creating a new and steadily growing patient population of adults with congenital heart disease (ACHD) [1,2]. The care for this expanding population, representing over 1.5 million patients in the USA alone, has emerged as a new subspecialty of cardiology [3].

This important increase in life expectancy of patients has generated new challenges, including arrhythmias that represent one of the main late complications [4]. Abnormal anatomy, post-surgical scarring, hemodynamic conditions and other systemic factors lead to build up a unique substrate for arrhythmia development. Reentrant tachycardias are by far the main mechanism encountered. Suture lines, patches or prosthetic material provide a core of inexcitable tissue that creates a central area of block, with the potential for reentrant circuits to form around these obstacles. Circuits encountered vary according to the anatomic defect and type of surgical repair but cavo-tricuspid isthmus-dependent circuits remain the most common [5]. Incisional intra-atrial reentrant tachycardia (IART), in particular around a right lateral atriotomy, is the second most common circuit. In other forms of CHD, such as in patients with old-style Fontan surgery (i.e., right atrium to pulmonary artery connections), long-term hemodynamic stress results in markedly abnormal atrial myocardium prone to various IART circuits around scar areas scattered in the atria. Furthermore, a substantial proportion of arrhythmias encountered have a focal activation pattern and are thought to be micro-reentrant circuits by virtue of their mode of induction and termination and response to pacing maneuvers. The term non-automatic focal atrial tachycardia (NAFAT) is commonly used in this setting to distinguish these arrhythmias from the more standard focal arrhythmias that are due to abnormal automaticity.

Discouraging experiences with pharmacological antiarrhythmic therapy in ACHD and recent advances in ablation techniques have resulted in a preference for interventional approaches [6], and catheter ablation has been now presented as a first-line therapy in international guidelines [7,8]. The improvement of ablative technologies, namely irrigated radiofrequency, contact force catheters, and 3D/high density mapping systems, has been associated with a significant improvement of outcomes [9]. However, the heterogeneity and complexity of phenotypes encountered require these cases to be performed by highly experienced operators, in specialized centers with multidisciplinary competencies. A thorough knowledge and understanding of anatomic specificities, vascular access issues, and main circuits encountered according to underlying phenotype is essential. In this review, we summarize those different points in the most common or distinctive defects to offer a didactic overview of atrial flutter catheter ablation in ACHD for the non-specialist electrophysiologist.

## 2 Tetralogy of Fallot

**Fig. 1:**
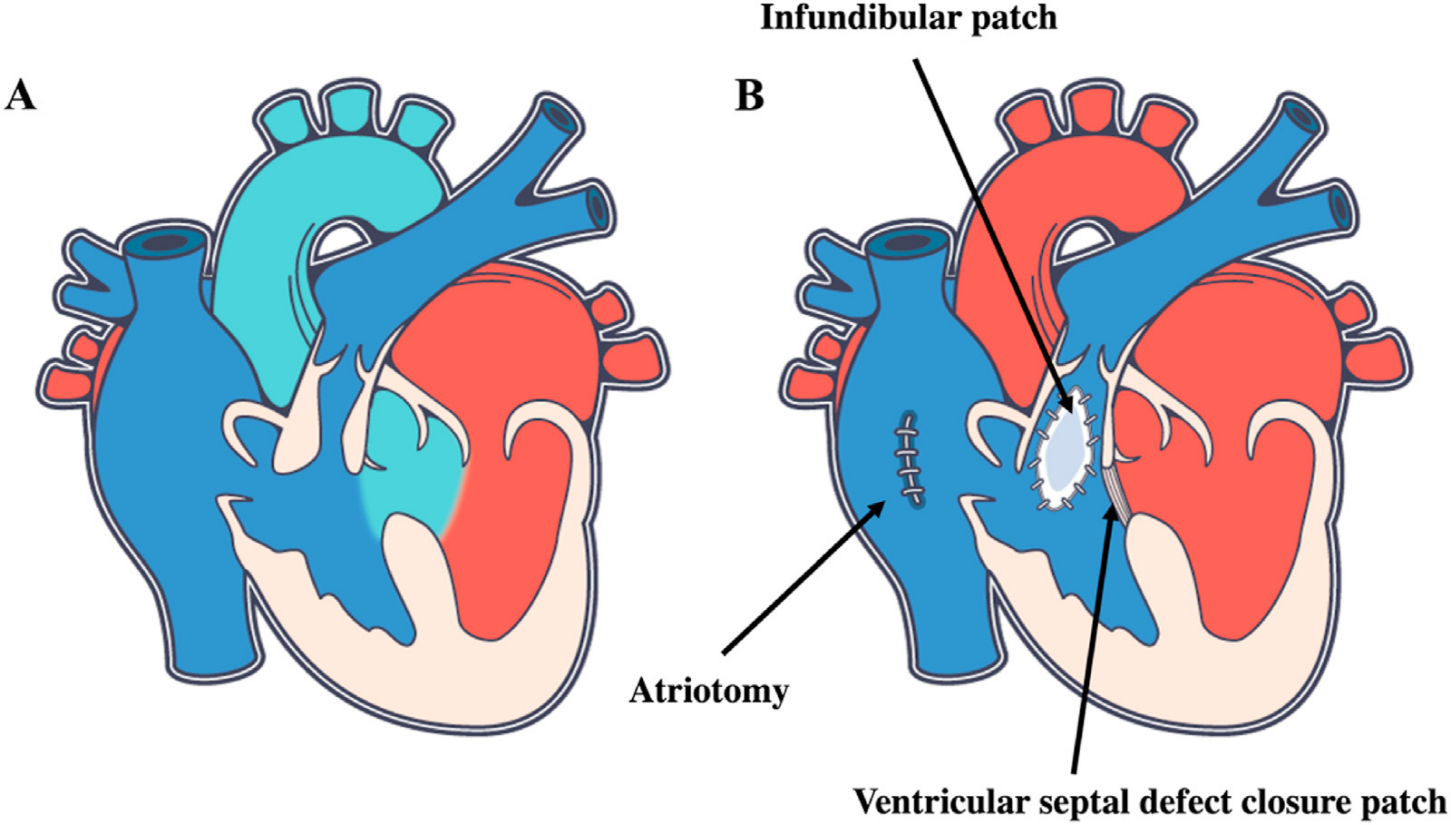
Tetralogy de Fallot before (A) and after (B) surgical repair.

Tetralogy of Fallot (TOF), representing 7–10% of all congenital heart diseases, is the most common cyanotic heart defect. First described in 1671 by Niels Stenson, this condition became known as the tetralogy of Fallot after further description by Etienne-Louis Fallot in 1888, a French pathologist. The four characteristic features of TOF are right ventricular (RV) outflow tract obstruction, ventricular septal defect, aortic override and RV hypertrophy. In the usual form, without other associated anomalies, surgical repair nowadays can be performed during the first year of life, sometimes after an initial palliative systemic-pulmonary shunt (Blalock-Taussig anastomosis, usually between the subclavian and the pulmonary artery) (Fig. 1). Initial “corrective” surgical approaches involved a large ventriculotomy with wide resection of the pulmonary infundibulum and frequently a transannular patch (from the pulmonary infundibulum to the pulmonary artery across the valve), and ventricular septal defect closure. Significant pulmonary regurgitation was almost invariably created. Further developments included transatrial and transpulmonary artery approaches, allowing for better preservation of the pulmonary valve function and less scarring on the RVOT musculature compared to previous access via a ventriculotomy.

**Fig. 2:**
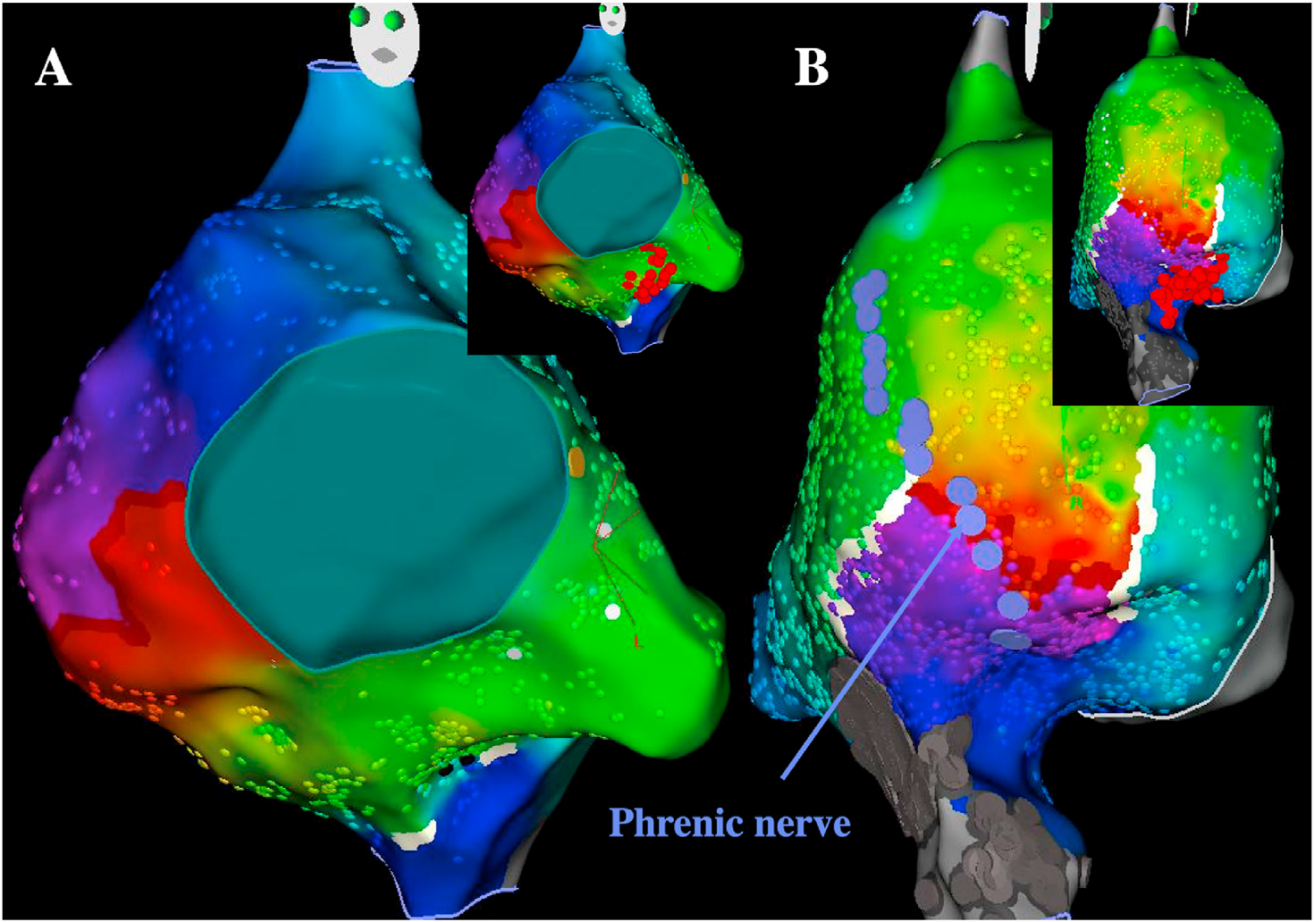
**Main atrial flutter circuits in patients with tetralogy of Fallot**. A: Cavotricuspid isthmus-dependent atrial flutter (left anterior oblique view). B: IART around atriotomy incision scar (lateral view) The yellow dot denotes the His signal, purple dots denote the phrenic nerve capture, red dots denote RF applications in the cavotricuspid isthmus and between the atriotomy scar and the inferior vena cava avoiding the phrenic nerve.

In patients with TOF referred for catheter ablation of atrial flutter, circuits encountered are quite reproducible. Cavotricuspid isthmus-dependent flutter is the main mechanism [10,11]. The second most common isthmus involves the lateral right atrial wall most of the time between scar tissue related to prior atriotomy incisions and the inferior vena cava. IARTs involving cavotricuspid isthmus, atriotomy incision, or both represented 51 of 52 atrial arrhythmias targeted in one study and 85% of circuits among 95 IARTs in another one (Fig. 2) [10,11]. Considering these relatively homogeneous and predictable circuits, acute success rates >90% are achieved. It is however important to carefully identify the course of the phrenic nerve before applying radiofrequency (RF) on the lateral right atrium wall to prevent phrenic nerve palsy and to continuously monitor phrenic nerve capture during RF delivery. The phrenic nerve is tagged during pacing with high output by moving the ablation catheter on the lateral wall from superior to inferior vena cava with optimal contact. As phrenic nerve mapping can result in atrial flutter termination, it should be performed at the beginning of the procedure in patients with sinus rhythm or after the IART circuit has been determined by electroanatomical mapping by stimulating at a relatively slow pace. Recurrences remain however relatively common during long-term follow-up of these patients, and while the mechanism is different from the atrial arrhythmia initially targeted in most cases, the circuit still involves the same potential isthmuses in most patients. Considering these observations, some operators perform a systematic prophylactic ablation line between the atriotomy scar and the inferior vena cava in patients with cavotricuspid isthmus-dependent flutter and a systematic prophylactic cavotricuspid isthmus ablation in patients with lateral right atrium wall-dependent IART. Although preliminary results have suggested the importance to consider different possible isthmuses in a series of 29 postcardiac surgery patients demonstrating that multiple ablation lines that targeted both cavotricuspid isthmus and scar-related isthmus were associated with better outcomes [12], the benefit associated with this approach has not been evaluated in this specific population so far.

## 3 Transposition of the Great Arteries with atrial switch

D-Transposition of the Great Arteries (D-TGA) accounts for 5% of all congenital cardiac malformations, and is characterized by the aorta originating from the right ventricle and the pulmonary trunk originating from the left ventricle. This anomaly is incompatible with survival in the absence of some form of communication between the systemic and pulmonary circulations. Immediate management of TGA in infancy involves preservation of the open ductus arteriosus or the creation of an atrial septal defect by balloon atrial septostomy (Rashkind maneuver), in order to maintain or create a communication between the two separate circulations. Currently, an arterial-switch operation is performed during the first days of life (connecting the aorta to the left ventricle and the pulmonary trunk to the right ventricle with corresponding coronary reimplantation), with excellent outcomes. However, before the mid-1980s, this anatomic repair was not an option. Instead, a physiologic repair was employed, meaning the construction of a “baffle” within the atria to direct systemic venous blood across the mitral valve into the left ventricle and the pulmonary venous blood across the tricuspid valve into the RV (atrial switch). The Senning operation was first introduced in 1959 to redirect blood via a relatively complex construction of autologous atrial tissue, whereas in 1964, Mustard proposed an alternative technique that used a pericardial patch. Thus, a physiologic circulation was restored, although the right ventricle continued to support the systemic circulation. Although arterial-switch surgery is currently the procedure of choice, most adults with D-TGA have had intra-atrial baffle repairs.

**Fig. 3:**
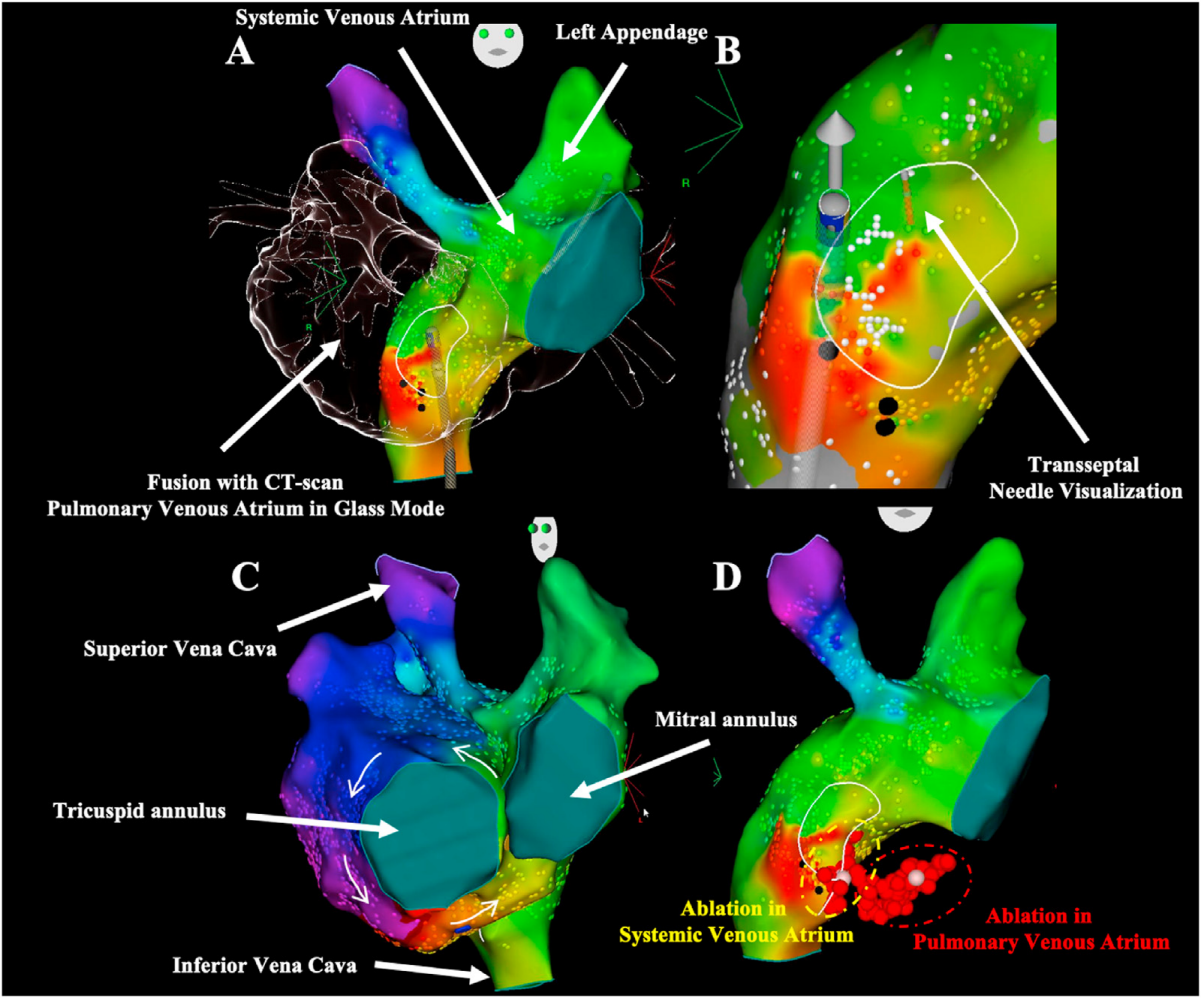
**Transbaffle puncture guided by 3D anatomical mapping and cavotricuspid isthmus ablation in a patient with D-TGA and atrial switch operation**. A: 3D anatomical reconstruction of the systemic venous atrium (subpulmonary) and fusion with the computed tomography (CT) scan. Note that a reference catheter is positionned in the left atrial appendage directly from the inferior vena cava in the systemic venous atrium B: The optimal site for transbaffle puncture is located and the transseptal needle is advanced and localized by the CARTO® system C: The final activation mapping identifies an atrial flutter around the tricuspid annulus D: Ablation (red dots) in both atrium (on each side of the baffle).

In patients with D-TGA and atrial switch operation, the coronary sinus usually drains in the pulmonary venous atrium but the left atrial appendage (directly accessible from the systemic venous atrium due to the surgery) provides a stable site to house the reference catheter that is reproducibly accessible in the event of inadvertent dislodgement. Contrast angiography via a pigtail catheter is sometimes performed in some centers to provide a greater degree of anatomic detail and to reveal potential baffle obstructions or shunts. While cavotricuspid isthmus-dependent flutters are also by far the main mechanism observed in those patients [[13], [14], [15]], it is actually partitioned in two with the tricuspid valve on the pulmonary venous side and inferior vena cava on the systemic venous side [16]. The relative proportion of the isthmus on each side of the circulation depends on the surgical variant but imposes ablation in both atria after transbaffle puncture or via retrograde approach. The transbaffle puncture has the potential to cause complications like pericardial effusion or tamponade besides the theoretical risk of damaging the intraatrial baffle creating a shunt between the two atria. It should be noted that the transbaffle puncture may cross atrial tissue in the case of a Senning baffle, but will cross either pericardium or synthetic material (e.g., Dacron or Goretex) in the case of a Mustard baffle, depending on the details of the operation. The puncture is however most of the times feasible and safe in those patients as the systemic venous atrium is surrounded by the pulmonary venous atrium. Transbaffle puncture can be safely performed without echocardiography guidance by using fusion of CT-scan 3D reconstructions and electroanatomical mapping images (Fig. 3) [17]. The main challenge of the retrograde approach is the sharp angle that the ablation catheter has to take to reach the whole cavotricuspid isthmus with sufficient contact (video 2). The retrograde approach is mainly used in centers where remote magnetic navigation systems are available or in rare cases of transbaffle failure. Despite these anatomical features, complete cavotricuspid isthmus block is usually achieved in most patients without particular difficulty. Other IART encountered are relatively heterogeneous and include circuits around the atriotomy or the posterior anastomosis (vertical atrial incision adjacent to the right pulmonary veins along the interatrial groove done during Senning surgery to channelise blood from the pulmonary veins around the baffle to the pulmonary venous atrium) in the pulmonary venous atrium [15]. Examples of cavotricuspid isthmus-dependent atrial flutter catheter ablation by transbaffle puncture (video 1) or retrograde aortic approach (video 2) are provided as supplementary material.

## 4 Atrioventricular septal defect

Atrioventricular septal defect (AVSD), also referred to as atrioventricular canal or endocardial cushion defect, covers a spectrum of congenital heart malformations characterized by a common atrioventricular junction coexisting with deficient atrioventricular septation. In partial AVSD (ostium primum atrial septal defect [ASD] and “cleft” left AV valve) there are separate atrioventricular valve orifices despite a common junction, while in complete AVSD the valve itself is also shared (up to 5 leaflets) with a variable deficiency of the inlet ventricular septum [18]. When the ventricular septal defect is restrictive, the term intermediate AVSD is used. Most patients will undergo surgical repair in childhood, which consists of closing intracardiac communications with patches and construction of two separate and competent atrioventricular valves, although some may have received palliation with pulmonary artery bands. Adults with complete AVSD not repaired in childhood will have developed pulmonary vascular disease with Eisenmenger physiology. Occasionally, patients with partial or intermediate AVSDs present in adulthood with unrepaired defects and symptoms related to heart failure, pulmonary vascular disease, or arrhythmias. Patients with unbalanced AVSD usually undergo single-ventricle palliation.

As in all patients with CHD, pre-procedural planning should include a comprehensive assessment of cardiac anatomy and a thorough review of prior surgical interventions. For example, in some cases, the patch used to close the ostium primum ASD is sewn with the coronary ostium retained on the left atrial side to limit the risk of AV node injury [19]. This information is important to note and could prevent frustrating attempts to cannulate a coronary sinus ostium that is not accessible from the right atrium. A thorough understanding of the conduction system in patients with AVSD is also of paramount importance to the safety and success of catheter ablation procedures. The major anatomic abnormalities of the AV conduction system are (i) postero-inferior displacement of the AV node; (ii) relatively short distance between the AV node and the origin of left bundle branching; (iii) marked postero-inferior displacement of the left bundle branch system; and (iv) relative hypoplasia of the anterior left bundle branches [20]. The posteroinferior displacement of the AV node and relative hypoplasia of the left anterior fascicle result in early impulse propagation to the posterior aspect of the ventricular septum and left ventricle, giving rise to the distinctive ECG pattern consisting of a superior QRS axis (leftward or extreme rightward), predominant S waves in the inferior leads, and R waves in aVL and aVR [21]. The AV node in patients with AVSD is displaced outside Koch's triangle. Indeed, the more deficient the atrioventricular septum, the more posteriorly deviated is the AV node. It is found within the apex of a second triangle delimited by the posterior insertion of the bridging tendon, the posterior fibrous area, and the posterior attachment of the posterior bridging leaflet, typically just anterior to the ostium of the coronary sinus [22]. The location of the AV node can also vary in relation to the coronary sinus, as a consequence of the extent of deficiency of the atrioventricular septum. When the atrioventricular septum is well formed, the coronary sinus is at some distance from the apex of this triangle, but when it is significantly deficient, the coronary sinus is adjacent to the apex and the AV node is close to its ostium [22].

**Fig. 4:**
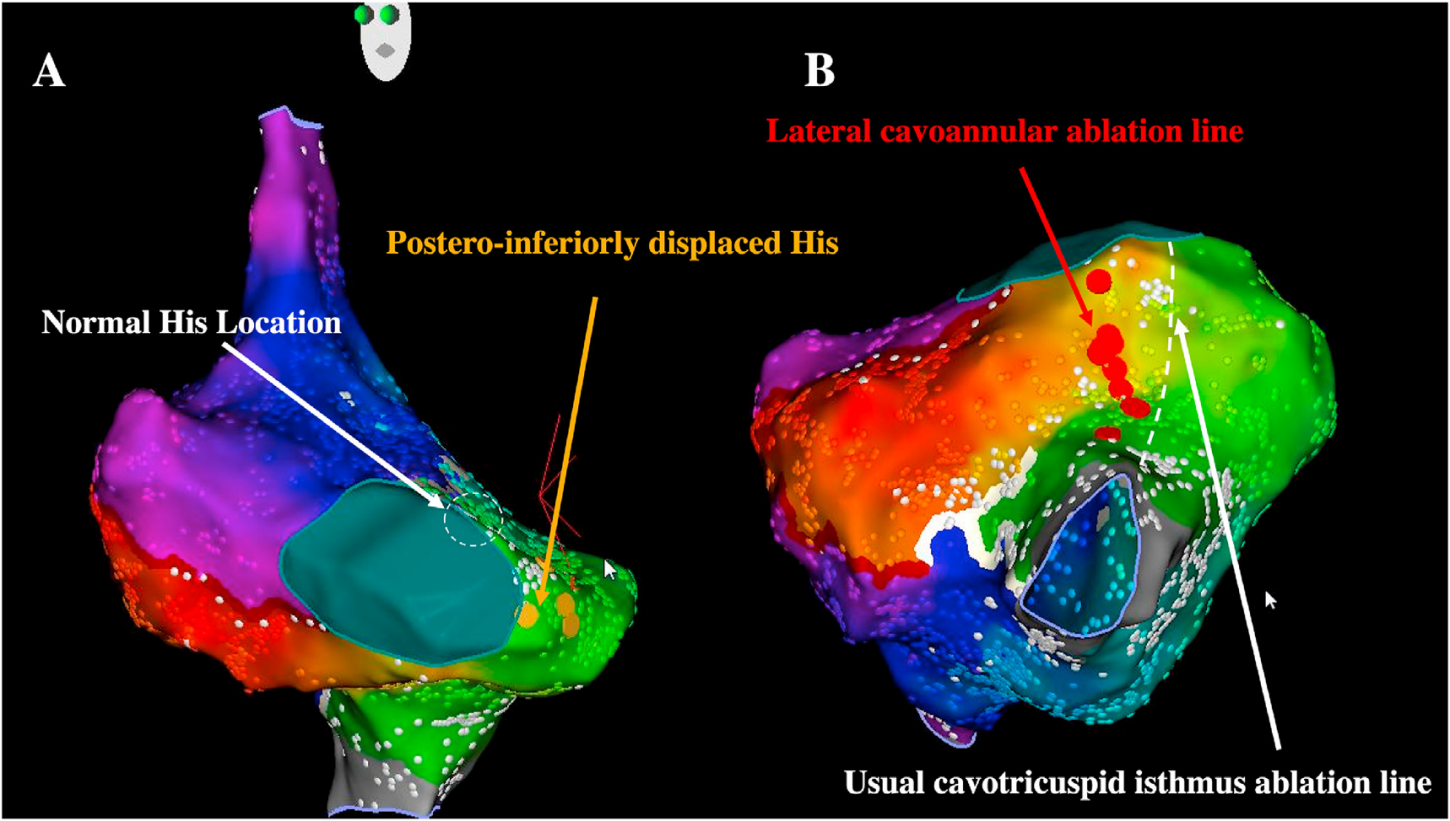
**Right-sided cavo-annular isthmus-dependent atrial flutter catheter ablation in a patient with AVSD**. Yellow dots denote the postero-inferiorly displaced His signal. Red dots denote RF applications with a relatively lateral ablation line between the inferior vena cava and the annulus. LAO (A) and LAO inferior (B) views.

**Fig. 5:**
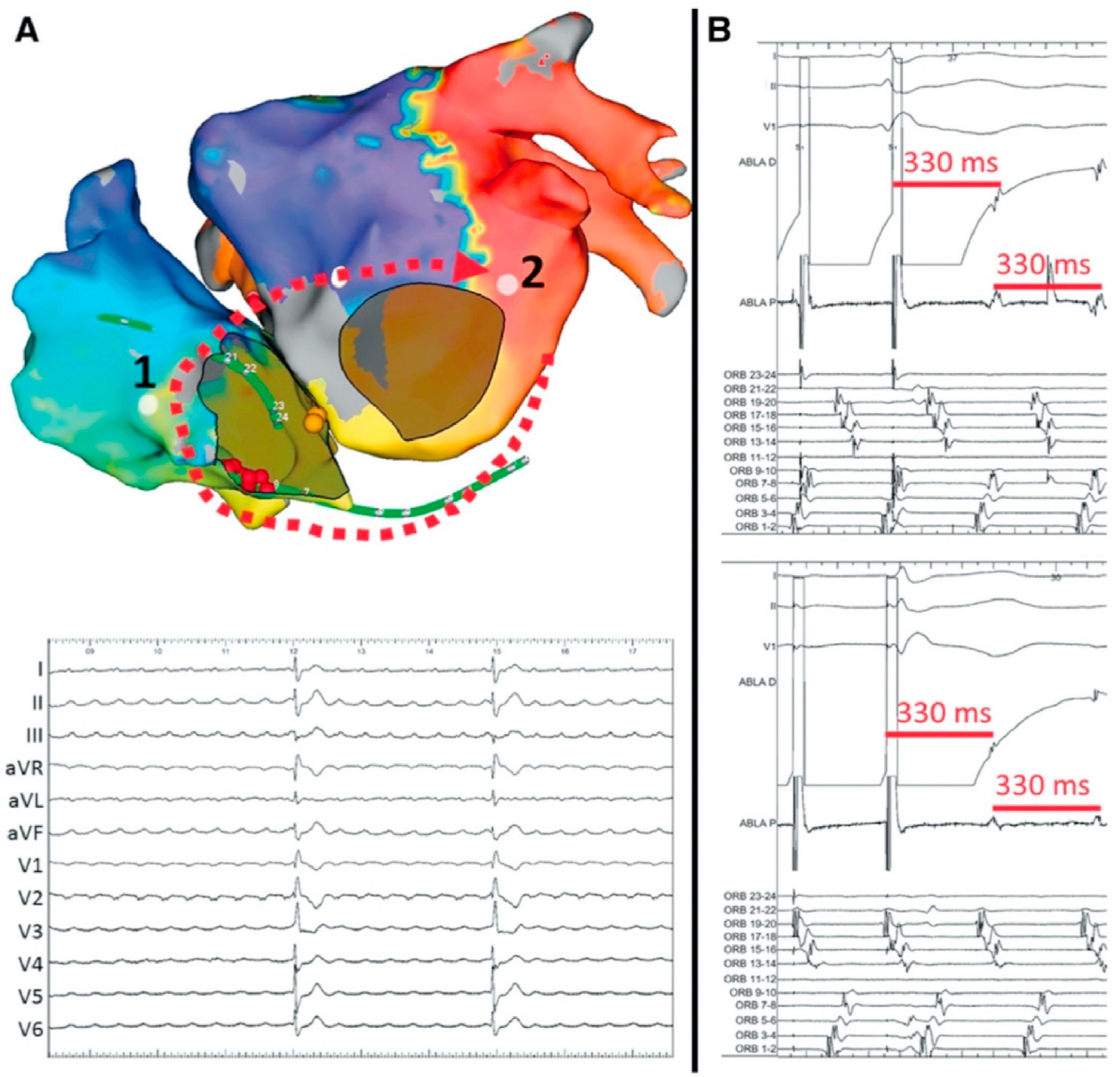
**Biatrial IART around the common AV valve annulus in a patient with AVSD**. Biatrial activation map (A) and concealed entrainment with post-pacing intervals on both atrium (B). Ablation is performed laterally along the right-sided cavo-annular isthmus (red dots). The yellow dots indicate the position of the inferiorly displaced His bundle Reproduced with permission from Moreno J et al. Europace 2017; 19:1438 [24].

Beyond typical IART dependent on the right-sided cavo-annular isthmus, other described circuits have predominantly been limited to case reports. Prior to ablating the right-sided cavo-annular isthmus, the His potential associated with the postero-inferiorly displaced AV node must be carefully identified. A more lateral cavo-annular ablation line in patients with AVSD than for the usual cavotricuspid isthmus in structurally normal hearts is very important in order to minimize the risks of AV block (Fig. 4). One must similarly be mindful of the posteriorly displaced His in the rare cases of atrioventricular nodal reentrant tachycardia ablation in these patients. Since patients with AVSDs can also have a single AV valve, biatrial IART circuits around the common AV valve have been described [23,24] (Fig. 5). In such cases, the arrhythmia can usually be terminated by a single ablation line between the inferior vena cava and the annulus (placed laterally to avoid AV block). Furthermore, patients with AVSDs frequently undergo one or more reintervention on the left AV valve. A surgical transseptal approach is commonly used to provide access to the left AV valve and is associated with scarring that may create a septal substrate for reentry. In particular, different types of biatrial IART circuits have been described post-mitral valve surgery using the transseptal approach that are dependent on interatrial connections at the posteroinferior part of the atrial septum, Bachmann's bundle, the fossa ovalis, and/or the coronary sinus ostium as interatrial bridges [25]. In such patients, posteroinferior interatrial connections (usually posterior and superior to the coronary sinus ostium) act as the critical limb for most circuits that require ablation from the bilateral insertion site.

## 5 “Univentricular” hearts and fontan surgery

The “Univentricular” heart represents a wide range of heterogeneous congenital heart defects characterized by a single functional ventricle and a second rudimentary or hypoplastic ventricular chamber. Although the most common form of univentricular heart is hypoplastic left heart syndrome, the most prevalent defect in adults is currently tricuspid atresia (absence of tricuspid orifice, hypoplastic RV and interventricular communication. Early management includes atrial septostomy (Rashkind maneuver) and/or a systemic-pulmonary shunt to maintain pulmonary blood flow. An atrio-pulmonary (first described by Fontan in 1971) or most recently total cavopulmonary anastomosis is then performed between the ages of 1 and 4 years, resulting in separated pulmonary and systemic circulations. The classic Fontan involved an anastomosis between the right atrial appendage and the main pulmonary artery, but with time leads to major right atrial dilatation, with consequent arrhythmias, thrombosis and right heart failure [26]. The modified Fontan approach now involves an anastomosis between the superior vena cava and the right pulmonary artery (Glenn anastomosis) and either an intra-atrial lateral tunnel (ILT) or more recently an extra cardiac conduit (ECC) connecting the inferior vena cava to the pulmonary artery [27].

**Fig. 6:**
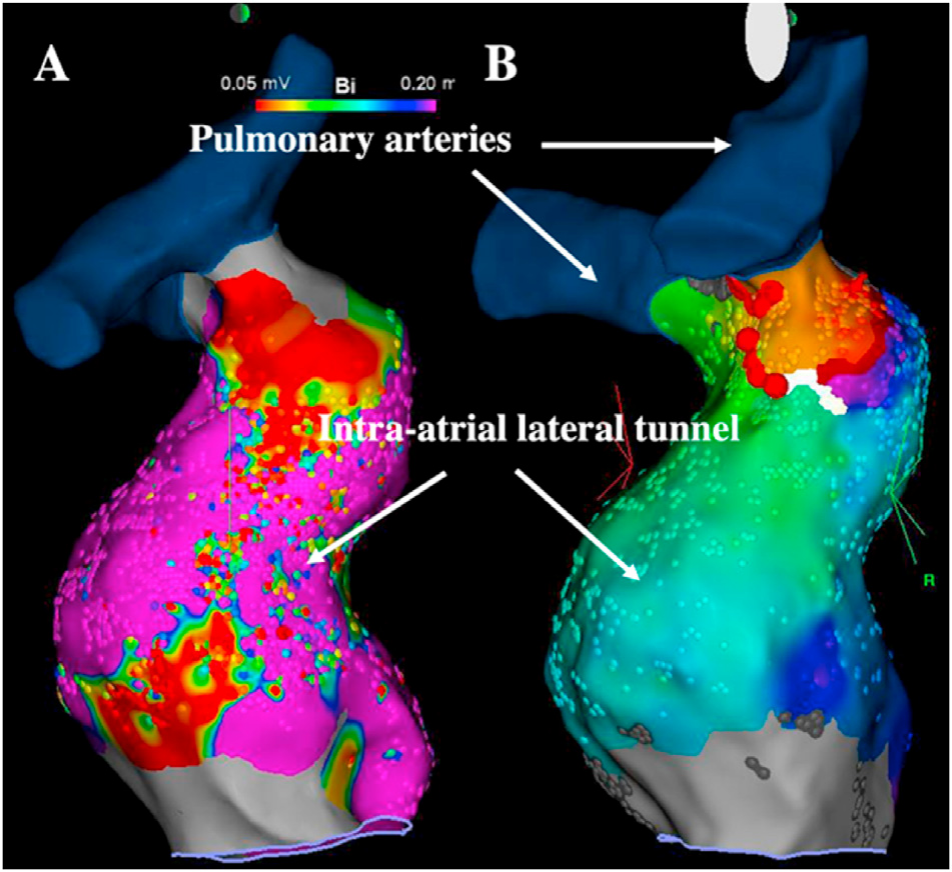
**Atrial flutter catheter ablation in a patient with intra-atrial lateral tunnel**. Voltage (A) and activation (B) mapping during atrial flutter ablation in a patient with Fontan circulation and intra-atrial lateral tunnel (lateral view). The whole circuit is located in the lateral tunnel around a scar area of the native superolateral right atrium wall. The ablation line (red dots) was successfully performed between the scar and the pulmonary artery.

The catheter ablation of atrial flutters in patients with Fontan circulation is particularly challenging due to the combination of complex and heterogeneous substrates for arrhythmia along with vascular access issues in patients with total cavopulmonary connections. The importance of atrial dilatation and remodeling results in diffuse abnormalities of atrial myocardium associated with multiple and hardly predictable circuits. In particular, in patients with old-style Fontan surgery (i.e., right atrium to pulmonary artery connections), long-term hemodynamic stress often results in markedly abnormal atrial myocardium prone to various circuits around scar areas scattered in the atria [[28], [29], [30]]. In patients with extracardiac conduits, circuits encountered are more reproducible. Most flutters involve a critical isthmus between the atrioventricular valve annulus and the over-sewn inferior vena cava, equivalent to the cavotricuspid isthmus in normal hearts [31]. Cavotricuspid isthmus-like-dependent IARTs have even been reported in patients with tricuspid atresia between the atretic annulus and the over-sewn inferior vena cava [32], although most of the critical isthmuses in these patients have been reported to be related to other mechanisms or circuits [28]. While critical isthmuses are sometimes confined in the intra-atrial lateral tunnel in patients where the native lateral atrium wall is part of the tunnel (Fig. 6), transtube or retrograde aortic approach is always required in patients with extracardiac conduits.

**Fig. 7:**
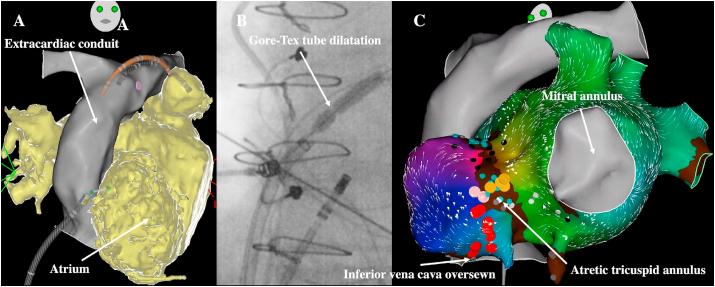
**Atrial flutter catheter ablation in a patient with extracardiac Fontan surgery**. A: 3D anatomical reconstruction of the extracardiac conduit (gray) and merging with the computed tomography (CT) scan (right atrium in yellow) to locate the optimal puncture site (right anterior oblique view). A reference decapolar catheter is inserted in the left pulmonary artery B: *Trans*-Gore-Tex tube puncture with balloon expansion to introduce the sheath C: Biatrial activation mapping using high-density catheter (Pentaray, Biosense) revealing a macro IART around the atretic tricuspid annulus with linear ablation performed between the atretic tricuspid annulus and the scar where the inferior vena cava was oversewn (left anterior oblique view). Yellow dots denote His signal, red dots denote RF applications.

Atrial pacing and atrial activity recording can usually be achieved in the intra-atrial tunnel, in the extra cardiac conduit (most of the time at the junction with the inferior vena cava), or in the left pulmonary artery that abuts the atrium and that allows to keep the catheter in a stable position. Another possibility is to insert a catheter through the oesophagus to record activity and pace the atrium [33]. In patients with a fenestration between the tube and the atrium, the introduction of catheters is only possible in a minority of cases due to its frequent involution or insufficient size but should be attempted. An angiography of the tube can be performed at the beginning of the procedure to look for, precisely locate the fenestration, and assess its size. The retrograde aortic approach is the safest strategy in centers where remote magnetic navigation systems are available, but transtube puncture has also demonstrated to be safe. Fusion of pre-procedural imaging and electroanatomical mapping images is essential, and some operators perform transtube accesses without echocardiography guidance (Fig. 7) [17]. A balloon expansion of the puncture site is most of the time required to get access to the atrium with the sheath. In patients with significant cavoatrial overlap, the transcaval puncture approach has recently been associated with a shorter time to pulmonary venous atrium in a single-center study [34]. Another example of IART catheter ablation in a patient with Fontan circulation (video 3) is provided as supplementary material.

## 6 Ebstein anomaly and tricuspid valve anomaly

Ebstein anomaly (EA) accounts for less than 1% of all congenital heart disease, but milder forms may go undiagnosed [35]. First described on autopsy by Wilhem Ebstein in 1866, EA is characterized by a malformed and apically displaced tricuspid valve. A portion of the RV is “atrialized”, i.e., functionally integrated within the right atrium. The consequence of EA is tricuspid regurgitation of varying severity (depending on the degree of displacement and the functional status of the tricuspid-valve leaflets), resulting in right atrial and RV enlargement. An interatrial communication is present in more than 80% of patients (atrial septal defect or patent foramen ovale). When possible, surgical repair versus valve replacement should be considered. Different techniques of tricuspid valvuloplasty involving various modifications of plication or resection of the atrialized ventricle have been proposed.

**Fig. 8:**
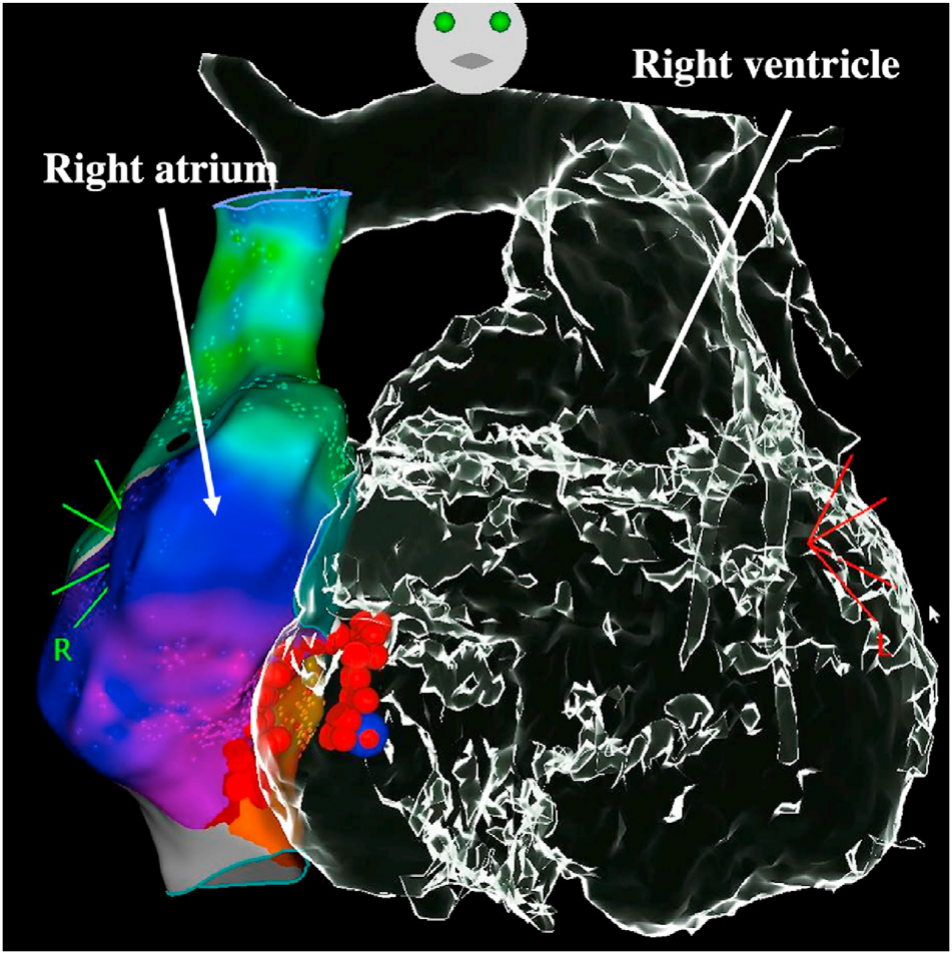
**Cavotricuspid isthmus-dependent flutter catheter ablation in a patient with Ebstein anomaly and tricuspid valve replacement (biological)**. Red dots denote RF applications on the cavotricuspid isthmus with applications required on the ventricular side to restore sinus rhythm and achieve bidirectional block. Flutter termination was achieved at the blue point location.

The dilated right atrium in EA creates a fertile environment for atrial arrhythmias of various sorts [36]. IART is the most common mechanism observed with cavotricuspid isthmus-dependent circuits that predominate or circuits around atriotomy incisions or septal patches in postoperative patients [37,38]. Catheter ablation should be considered before surgery in most cases, particularly in symptomatic patients, but systematic electrophysiologic study is also proposed by some teams in patients with no prior suspicion of arrhythmias to target underlying arrhythmias substrate by catheter ablation or to guide prophylactic surgical cryoablation (mainly accessory pathways) [39]. It is important to emphasize that any subsequent catheter ablation procedures along the tricuspid annulus will be complicated if an annuloplasty ring was included as part of the repair or in case of tricuspid valve replacement. Prosthetic valve or annuloplasty ring along or near the tricuspid annulus may result in inaccessible atrial tissue that is part of the critical isthmus. Furthermore, the potential risk of damaging the prosthetic valve must be considered. A recent multicenter study including patients with CHD demonstrated that the acute success for annular substrates was lower in patients with tricuspid valve replacement or ring (73%) compared to patients with tricuspid valve repair (92%) and to patients with no surgery (94%) [40]. Another study demonstrated that ablation on the ventricular size of the prosthesis may be required in a significant proportion of cases to achieve cavotricuspid isthmus bidirectional block (Fig. 8) [41]. A unique case using a transseptal system to gain access to protected myocardium below a prosthetic tricuspid valve has even been reported [42]. Focal atrial tachycardia has also been encountered in EA patients but is usually observed as a secondary arrhythmia that is uncovered during ablation for accessory pathway or IART circuits. Last, for some patients with long-standing cyanosis and right-to-left-shunting, the left atrium can become significantly enlarged as well, and left atrial arrhythmias and atrial fibrillation can become a recurring issue.

## 7 Congenitally Corrected Transposition of the Great Arteries (double discordance)

Congenitally Corrected Transposition of the Great Arteries (cc-TGA, also named as L-Transposition of the Great Arteries) is a rare defect (<1% of congenital heart disease), characterized by atrio-ventricular and ventriculo-arterial discordance. Ventricles and their attached valves are reversed, with a sub-aortic RV and a sub-pulmonary left ventricle. Patients with cc-TGA may remain asymptomatic and be diagnosed later in life, although associated anomalies (for example, severe tricuspid regurgitation, VSD and/or subpulmonary outflow obstruction) can be responsible for a much earlier presentation. The systemic atrio-ventricular valve is sometimes of Ebstein type with insertion abnormalities that account for a variable degree of intrinsic regurgitation. In the absence of significant associated anomalies, surgery or other intervention is rarely required and the course of this entity mainly depends on the function of the morphological RV which supports the systemic circulation. A “double switch” repair (or anatomic repair) associating atrial (Senning surgery) and arterial switches and that results in a systemic left ventricle is performed in some patients.

**Fig. 9:**
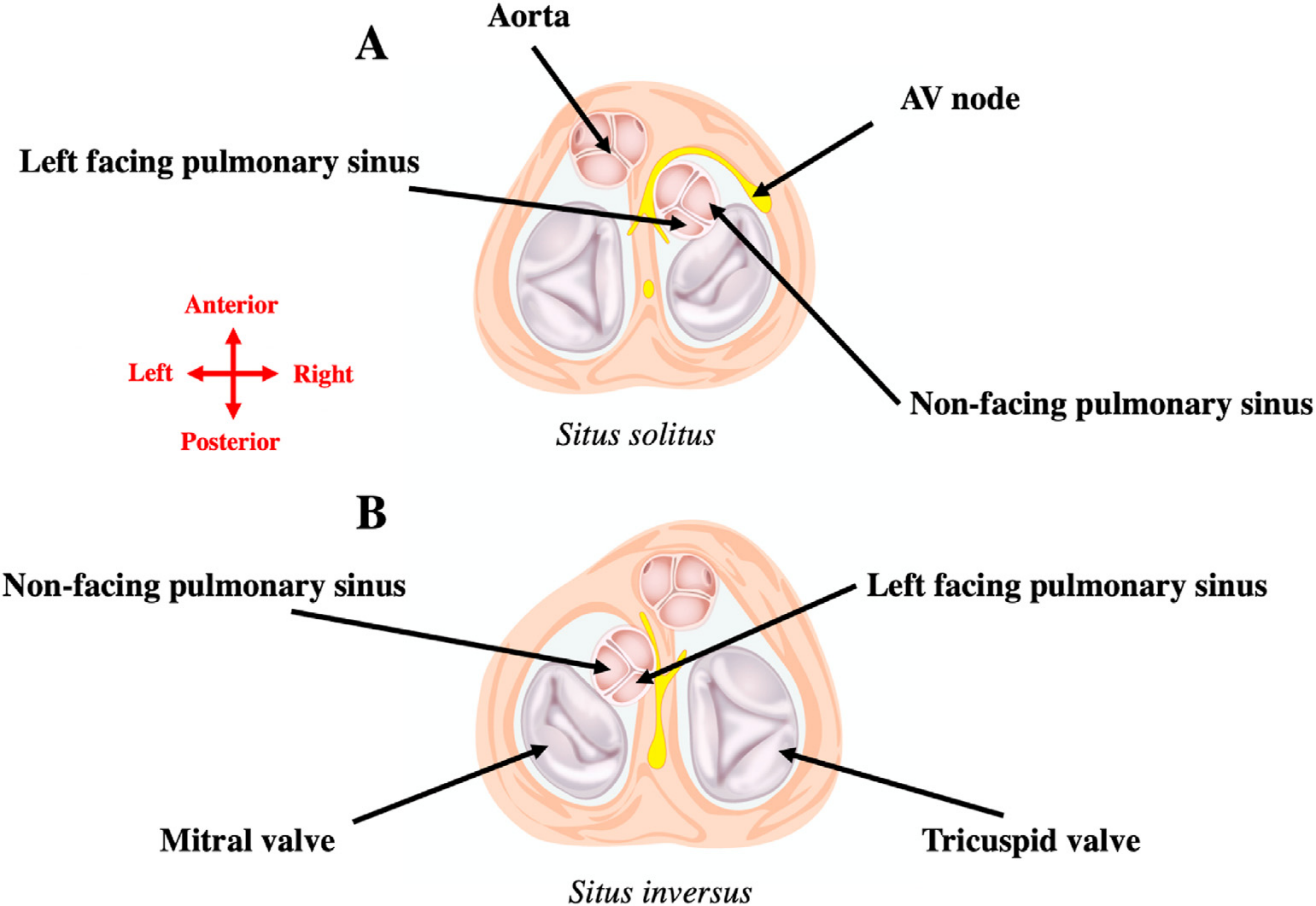
**Cardiac conduction system and relationship between pulmonary sinuses and atria in cc-TGA.** A: In patients with situs solitus, the AV node is displaced anterior and slightly more lateral below the orifice of the right atrial appendage close to the atrial aspect of the pulmonary-mitral continuity. B: In patients with situs inversus, the AV node is usually located in its normal position at the apex of the triangle of Koch. In both atrial arrangements, the non-facing pulmonary sinus is in contact with the right atrium and the left-facing is in contact with the left atrium.

Different anatomical specificities are of relevance in patients with cc-TGA. First, the cardiac conduction system location depends on atrial arrangement. In patients with situs solitus, the AV node is displaced, anterior and slightly more lateral, below the orifice of the right atrial appendage close to the atrial aspect of the pulmonary-mitral continuity. A second regularly situated posterior hypoplastic AV node is sometimes associated but does not conduct to the ventricles in most cases. By contrast, in patients with situs inversus, the AV node is usually located in its normal position at the apex of the triangle of Koch (albeit left-sided) (Fig. 9) [21,43]. Second, accessing the coronary sinus may be challenging in some patients as they may have abnormalities of the coronary sinus ostium including ostial atresia, multiple or displaced coronary sinus ostia, and coronary sinus draining to right atrium through multiple Thebesian veins [44].

**Fig. 10:**
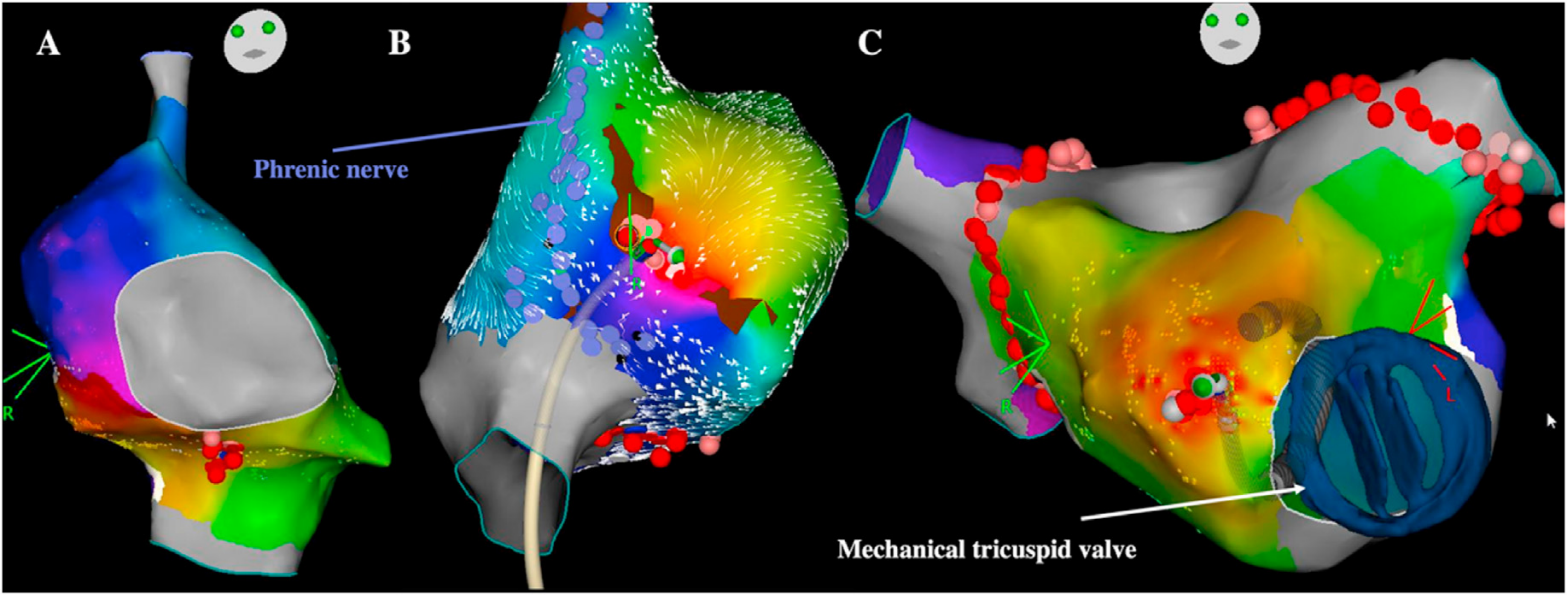
**Atrial flutters catheter ablation in a patient with cc-TGA and mechanical tricuspid valve**. A: A first atrial arrhythmia was induced with circuit around the mitral (right-sided) annulus with cavomitral isthmus ablation (left anterior oblique view) B: The critical isthmus of the second IART induced was on the lateral wall of the right atrium (right inferolateral view). The phrenic nerve projection is represented with purple dots C: The last arrhythmia induced was a left focal atrial tachycardia next to the mechanical tricuspid valve. Pulmonary vein isolation was also performed because the patient had atrial fibrillation history (anteroposterior view) Red dots denote RF applications. This case is illustrated in the video 4 provided as supplementary material.

Only a few experiences of atrial flutters catheter ablation have been published in patients with cc-TGA. Cavomitral isthmus (right-sided) -dependent circuits have been described as well as incisional IARTs around atriotomy incisions [45]. Furthermore, patients with cc-TGA frequently have significant tricuspid regurgitation associated with systemic right ventricular dysfunction and may undergo one or more reintervention on the tricuspid valve (left-sided). The chronic tricuspid valve regurgitation can result in progressive left atrial dilatation associated with the development of left atrial arrhythmias (Fig. 10 and (video 4) [[46], [47], [48]]. Lastly, as in patients with cc-TGA and situs solitus the pulmonary outflow tract, rather than the aortic root in normal hearts, is located deeply between the 2 atria, successful catheter ablation of atrial tachycardia has also been reported in pulmonary sinus [49]. The non-facing pulmonary sinus is in contact with the right atrium and the left-facing is in contact with the left atrium (Fig. 9). Another example of atrial flutter catheter ablation in a patient with cc-TGA and double switch repair (video 5) is provided as supplementary material.

## 8 Atrial septal defect

Atrial septal defect (ASD) accounts for 30% of congenital heart disease detected in adulthood. Three major types of interatrial communications are described: ostium secundum, ostium primum, and sinus venosus defects. The ostium secundum is the most prevalent and involves the region of the fossa ovalis. The ostium primum defect is part of the spectrum of AVSD, associated with a trileaflet (“cleft”) left AV valve. The sinus venosus defect is usually located at the junction of the right atrium and superior vena cava and is almost always associated with partial anomalous pulmonary venous return. A sizeable defect results in dilation of the right heart chambers, and may cause pulmonary hypertension, right heart failure, supraventricular tachycardia and paradoxical systemic embolism. Patients with symptomatic or hemodynamically significant ASD should be offered elective closure, irrespective of age. Percutaneous approach is usually preferred, but surgery is required for patients with ostium primum and sinus venosus ASDs, and for patients with ostium secundum defects when anatomy is unsuitable for device closure.

**Fig. 11:**
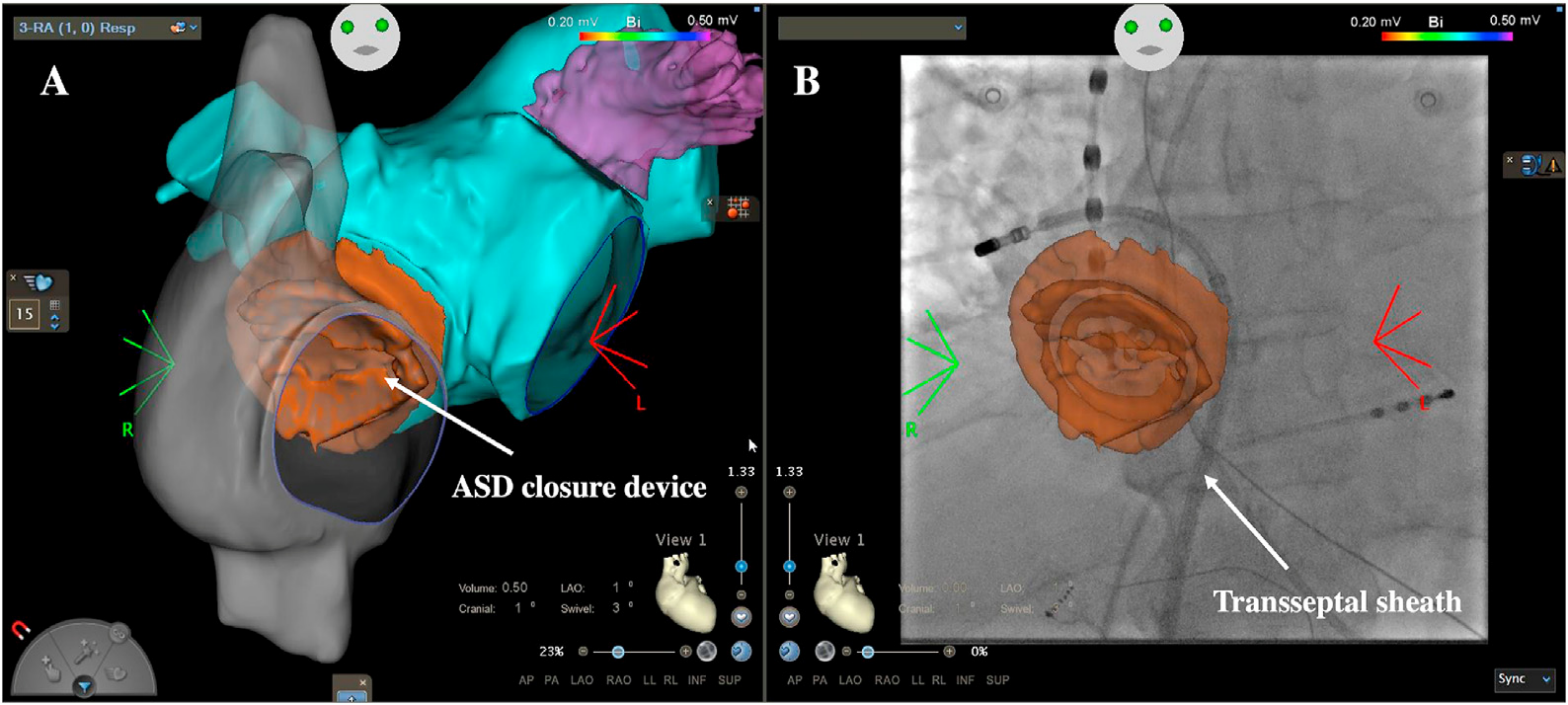
**Transseptal access in a patient with atrial septal defect closure device**. A: 3D anatomical reconstruction of the right atrium (gray) and merging with the computed tomography (CT) scan (left atrium in blue and ASD closure device in orange) to locate the optimal puncture site (anteroposterior view). B: Fluoroscopy view with superposition of the ASD closure device showing the transseptal sheath inserted just inferiorly to the device. The ablation catheter is placed in the right sided pulmonary vein.

The magnitude of the left-to-right shunt depends on the ASD size and the relative diastolic filling properties of the left and right ventricles, but results in progressive right atrial dilatation associated with an increased risk of right-sided atrial flutter. Although cavotricuspid isthmus-dependent flutters are predominant, circuits on the lateral right atrium around atriotomy or cannulation sites or around the septal closure patch or device can be encountered in repaired patients [5,29,50]. Left-sided atrial flutter and atrial fibrillation can also develop in unrepaired or late-repaired patients, with age at the time of correction being the factor most strongly associated with left arrhythmias [51]. While ASD closure, be it surgical or percutaneous, reduces the arrhythmia burden, a 4.1% annual incidence of new-onset atrial fibrillation was reported among 1062 patients with transcatheter ASD closure [52]. In those patients, different studies have reported that successful transseptal access can be safely achieved in more than 90% of cases under echocardiography or intracardiac echocardiography guidance [45,46]. The transseptal access is usually possible across a portion of the native septum, most of the time at the infero-posterior periphery of the device (Fig. 11). In patients with no areas of native septum suitable for transseptal access, direct puncture of the closure device may be required with the need for progressive upzising of the dilatator or balloon expansion to allow the transseptal sheath to cross the device (preferably at least 6 months after closure device implantation). Usually, no residual shunt is observed remotely from the ablation, including in patients with access through the device. Remote magnetic-guided catheter navigation offers an alternative means of accessing the left atrium by a retrograde aortic approach, as has been performed in a patient with an oversized ASD closure device [53]. In patients with newly diagnosed ASD and atrial arrhythmias, it is preferable to proceed with catheter ablation prior to ASD closure whenever feasible.

## 9 Technical issues

Electroanatomical mapping now appears as an indispensable tool to increase procedures safety and efficacy in complex defects. The 3D-image integration is also particularly valuable and a detailed preprocedural analysis enables to completely understand the anatomy and to outline a careful procedure plan, including best and alternative catheter access options. In cases of occluded veins due to vascular anomalies, prior interventions, or azygos continuation (or other systemic venous return anomaly), superior (jugular or subclavian) or in rare instances transhepatic access can be planned (video 5). Other creative methods such as transthoracic direct percutaneous access or surgical and interventional hybrid procedures have been described, but are best reserved for carefully selected cases. A perfect mastery of the different tools and techniques to perform transbaffle or transtube punctures in complex anatomies is also essential. The role of 3D-image integration is major in those cases, and the puncture can be safely performed after careful fusion of CT-scan or magnetic resonance reconstructions with electroanatomical mapping images to precisely identify the best site for puncture, with or without echocardiography guidance [17]. The transseptal needle and the guidewire can be connected to the mapping system to localize site of puncture towards increasing safety and decreasing fluoroscopy use (Fig. 3). The puncture can also be performed directly using a 0.014 very stiff guidewire inserted in the transseptal needle. Radiofrequency needle use and balloon expansion are sometimes required to insert the sheath through the baffle or the tube (Fig. 7). Remote magnetic-guided catheter navigation offers an alternative means of accessing the pulmonary venous atrium by a retrograde aortic approach in centers where this technology is available [54].

## 10 Conclusion

Catheter ablation of atrial flutters is by far the most common electrophysiological procedure performed in ACHD patients and the number of interventions is expected to continuously increase year after year. A thorough understanding and knowledge of phenotype-specific anatomic features and of main expected arrhythmia mechanisms or circuits according to underlying substrate is essential to address these challenging cases. When performed in expert centers, acute procedural success rates have significantly improved and are excellent, but recurrences remain a common issue, with different mechanisms or circuits frequently encountered. The impact of different ablation strategies on late recurrences are still not well defined and a lot of work remains to be done to improve long-term outcomes in these patients. Preliminary observational data have for instance suggested the interest of systematically targeting all inducible atrial arrhythmias, whether previously documented or not, beyond clinical arrhythmia termination [55]. A lot of hope and research is also based on the identification and prediction of arrhythmia substrate before arrhythmia development by imaging or electroanatomic mapping techniques using for example voltage or conduction velocity analysis to identify potential critical isthmuses and to deliver a prophylactic patient tailored ablation approach [[56], [57], [58]].

## Declaration of competing interest

None.
